# Humoral response among patients with interstitial lung disease vaccinated with the BNT162b2 SARS-Cov-2 vaccine: a prospective cohort study

**DOI:** 10.1186/s12931-022-02155-x

**Published:** 2022-09-01

**Authors:** Barak Pertzov, Einat Shmueli, Haim Ben Zvi, Amir Massarweh, Tamar Barkan, Asaf Ness, Yael Shostak, Lev Freidkin, Osnat Shtraichman, Mordechai R. Kramer

**Affiliations:** 1 Pulmonary Division, Rabin Medical Center, Beilinson Campus, 49100 Petach Tikva, Israel; 2 Pediatric Pulmonology Institute, Schneider Children’s Medical Center of Israel, Petach Tikva, Israel; 3 Microbiology Laboratory, Rabin Medical Center, Petach Tikva, Israel; 4 Davidoff Center, Rabin Medical Center, Petach Tikva, Israel; 5 Internal Medicine E, Rabin Medical Center, Petach Tikva, Israel; 6 Sackler Faculty of Medicine, Tel Aviv University, Tel Aviv, Israel

## Abstract

**Background:**

Patients with interstitial lung disease (ILD) are at high risk of severe COVID-19 infection. Additionally, their anti-inflammatory and antifibrotic treatment may cause immunosuppression. Nevertheless, their ability to mount an adequate immune response to messenger RNA SARS-CoV-2 vaccines was not evaluated. Therefore, we aimed to evaluate the humoral response after the BNT162b2 vaccine among idiopathic pulmonary fibrosis (IPF) patients treated with antifibrotic therapy and among non-IPF ILD patients treated with anti-inflammatory therapy.

**Methods:**

We conducted an observational prospective cohort study to evaluate the level of anti-spike (S-IgG) antibodies after two doses of the BNT162b2 vaccine in patients with ILD. The cohort included 40 patients with idiopathic pulmonary fibrosis (IPF) treated with anti-fibrotic therapy and 29 patients with non-IPF ILD treated with anti-inflammatory therapy. For S-IgG titer measurement, one serology test was drawn from all patients 4–6 months after the second vaccine dose. In addition a control group matched for age and sex was created from a healthy control cohort of 107 patients. The study was conducted in Rabin Medical Center (Israel) between June and August 2021.

**Results:**

All patients in the anti-fibrotic arm were seropositive (40/40), corresponding to the matched control group (P = 1.0). The anti-fibrotic arm had a significantly lower median antibody titer in comparison to the matched control group (361.10 [IQR, 207–811] AU/ml vs. 820.75 [IQR, 459–1313] AU/ml; P < 0.001). Only 48.3% (14/29) of patients in the anti-inflammatory arm were seropositive in comparison to 100% (29/29) in the healthy control group (P < 0.001). The anti-inflammatory arm had a significantly lower median antibody titer in comparison to the healthy control group (39.6 [IQR, 4.25–165] AU/ml vs. 970.1 [IQR, 505–1926] AU/ml; P < 0.001).

**Conclusion:**

IPF patients treated with antifibrotic therapy mount an adequate immune response after 2 doses of the BNT162b2 vaccine, and maintain a 100% seropositivity rate 4–6 months after vaccination. However, their antibody titer was reduced in comparison to a healthy control group. Among patients with non-IPF ILD treated with anti-inflammatory therapy, 48% were seronegative 4–6 months after the second vaccine dose. Moreover, treatment with rituximab caused significant immunosuppression, even in comparison to other anti-inflammatory treatments.

**Supplementary Information:**

The online version contains supplementary material available at 10.1186/s12931-022-02155-x.

## Introduction

Coronavirus disease 2019 (COVID-19), caused by severe acute respiratory syndrome coronavirus 2 (SARS-Cov-2), is an ongoing global pandemic [1]. Several vaccines are currently used for disease prevention, the most common are the mRNA vaccines. In contrast to healthy volunteers who mount an adequate immune response after vaccination, patients with chronic disease, who are treated with anti-inflammatory therapy have a reduced immune response [2–5]. Patients with interstitial lung disease (ILD) are treated with both anti-inflammatory and antifibrotic therapy in accordance with their underlying disease. Idiopathic pulmonary fibrosis (IPF), the most common of the idiopathic interstitial pneumonias, is a fibrotic disease treated solely with antifibrotic therapy. Two antifibrotic drugs are currently approved for IPF therapy, nintedanib, which is a tyrosine kinase inhibitor that exerts an inhibitory effect on fibroblast growth factor receptor (FGFR), endothelial growth factor receptor and platelet-derived growth factor receptor (PDGFR) [6, 7], and pirfenidone, which is an antifibrotic drug that reduces fibrosis in lung, hepatic, kidney, and cardiac tissue. Pirfenidone works partially by inhibition of transforming growth factor beta (TGF-β), however, its direct molecular target is unknown [8, 9]. The impact of antifibrotic treatment on the humoral response after mRNA vaccine have yet to be evaluated in clinical trials. Since patients with ILD are at higher risk of contracting COVID-19 and developing severe disease that results in significant morbidity and mortality, it is essential to evaluate these patients' immune response to the COVID-19 mRNA vaccine [10–12]. This trial aims to evaluate the humoral response after the BNT162b2 vaccine among IPF patients treated with antifibrotic therapy and among non-IPF ILD patients treated with anti-inflammatory therapy.

## Methods

We conducted an observational prospective cohort study at Rabin Medical Center between June to August 2021, to evaluate the humoral response to the BNT162b2 vaccine among patients with interstitial lung disease. Patients eligible for inclusion were adults (≥ 18 years) who were vaccinated with two doses of the BNT162b2 vaccine, diagnosed with idiopathic pulmonary fibrosis treated with anti-fibrotic therapy (nintedanib or pirfenidone) or non-IPF ILD patients treated with anti-inflammatory treatment (e.g., glucocorticoids, antimetabolites, and rituximab). Exclusion criteria were patients who were previously infected with COVID-19 (as documented by positive PCR nasal swab). All patients signed an informed consent form, and the study was approved by the institutional review board at Rabin Medical Center (RMC-0294-21).

### Data collection

Patients were invited to participated in the study when they arrived for their routine evaluation in the ILD clinic or by a public invitation to participate that was sent to all ILD patients in the country, with aid of the Israeli ILD patient foundation. Patients that agreed to participate and signed an informed consent form were invited to the pulmonary institute for serology testing. From each patient one serology test tube was drawn and sent to the lab for analysis. Relevant demographic and clinical data, including immunosuppressive drug regimens, vaccination dates and infection rates 6 months after the antibody level was analyzed, were recorded from the electronic medical records at Rabin Medical Center (RMC).

### Treatment arms

This observational study included two treatment arms and one control arm: an antifibrotic treatment arm included IPF patients and an anti-inflammatory treatment arm included non-IPF ILD patients. The control arm included healthcare workers from RMC and a cohort of healthy volunteers that were recruited as a control group for a similar study at RMC [13] and were also used as a control group in this study. For each evaluated arm a 1:1 age and gender matched control group was created from the healthy control cohort.

### Blood sample processing

Whole blood samples were drawn from participants at study visit. Serum was separated by centrifugation, aliquoted and stored at − 20 °C until the serological assay was performed.

SARS-CoV-2 IgG II Quant assay (Abbott Ireland Diagnostic Division) was performed using the ARCHITECT® i2000SR immunoassay analyzer in accordance with the manufacturer’s package insert. The assay is a chemiluminescent microparticle immunoassay (CMIA) used for the quantitative determination of immunoglobulin class G (IgG) antibodies to the receptor binding domain (RBD) of S1 subunit of the spike protein of SARS-CoV-2 (S-IgG) in human serum and plasma samples. In the assay, S-IgG antibodies bind to antigen coated paramagnetic microparticles, and anti-human IgG acridinium-labeled conjugate is added to create a reaction mixture. Following further processing, the resulting chemiluminescent reaction is measured as a relative light unit (RLU), with the detected RLU directly related to the amount of S-IgG in the sample. S-IgG titers of 50 AU/ml and greater in the immunoassay test are interpreted as positive [14–16].

### Outcomes

The primary outcome was to assess the rate of seropositivity in ILD patients following the BNT162b2 vaccine, as measured by S-IgG antibodies present 4–6 months after receiving two vaccine doses. Positive S-IgG antibody titer was defined as ≥ 50 AU/ml. Secondary outcomes were the median titer and geometric mean titer (GMT) of S-IgG antibodies, identification of independent predictive factors for negative serologic response by multivariate analysis and COVID-19 infection rates 6 months post antibody level analysis.

### Statistical analysis

Demographic and clinical baseline characteristics were compared with the Student's t-test, chi-squared test and Mann–Whitney U test, as appropriate. The primary outcome was analyzed with the chi-squared test. Antibody level was presented as median (IQR) and GMT and was analyzed with the Mann–Whitney U test and Student's t-test, respectively. Comparison between the antibody titer of different treatment groups (e.g., control, antifibrotic, antimetabolites + glucocorticoid and rituximab) was analyzed by LOG10 conversion followed by one way ANOVA with Bonferroni correction for multiple comparisons. Multivariate analysis for independent predictors of negative serologic response was conducted with linear regression. SPSS version 27 (IBM corp., Armonk, NY) was used for statistical analysis.

## Results

Overall, 176 participants were included in the final analysis. 40 patients in the anti-fibrotic arm, 29 patients in the anti-inflammatory arm and 107 patients in the control arm. Patients with IPF that were treated with anti-fibrotic therapy (anti-fibrotic arm) had a median (IQR) age of 71 (67–75) and 27 patients (67.5%) were men. The matched control group had a median (IQR) age of 69 (63–72) and 16 (40%) were men. All patients in the anti-fibrotic arm were seropositive (40/40), corresponding to the matched control group (P = 1.0). However, the antifibrotic arm had a significantly lower median antibody titer in comparison to the matched control group (361.10 [ IQR, 207–811] AU/ml vs 820.75 [IQR, 459–1313] AU/ml; P < 0.001). The median (IQR) time between the second vaccine dose and the serology test was 173 days (168–182) in the anti-fibrotic arm and 128.55 days (123–142) in the controls (P < 0.001) (Table 1).

**Table 1:** Baseline characteristics and antibody titers among patients treated with anti-fibrotic treatment and matched control

	Anti-fibrotic Treatment (n = 40)	Matched control (n = 40)	P value
Age, median (IQR), y	71 (67–75)	69 (63–72)	0.02
Male sex (%)	27 (67.5)	16 (40)	0.01
IgG titer, median (IQR), Au/ml	361.10 (207–811)	820.75 (459–1313)	0.001
IgG titer GMT (SD)	441.26 ± 3.15	835.41 ± 2.07	0.004
Seropositive (%)	40 (100)	40 (100)	1.0
Days post vaccination, median (IQR)	173 (168–182)	128.55 (123–142)	< 0.001

Patients with non-IPF ILD treated with anti-inflammatory treatment had a median (IQR) age of 67 (55–73) and 11 (38%) were men. The median (IQR) dose of oral corticosteroids and mycophenolate was 8 mg (5–8) and 2 g (1–2), respectively. Eight patients were treated with rituximab, the median (IQR) number of rituximab doses per patient was 3 (2–4) and the median (IQR) number of days post the last rituximab dose was 123 days (36–147). The matched control group had a median (IQR) age of 70 (56–72) and 11 (38%) were men. Within the anti-inflammatory arm, all patients (29/29) were treated with antimetabolites and/or oral corticosteroids, 8 patients were also treated with rituximab. Only 48.3% (14/29) of patients in the anti-inflammatory arm were seropositive in comparison to 100% (29/29) in the healthy control group (P < 0.001). The anti-inflammatory arm had significantly lower median antibody titer in comparison to the matched control group (39.6 [IQR, 4.25–165] AU/ml vs 970.1 [IQR, 505–1926] AU/ml; P < 0.001). The median (IQR) time between the second vaccine dose and the serology test was 179 days (168–184) in the anti-inflammatory arm and 137 days (122–158) in the controls (P < 0.001) (Table 2). When compared to the anti-inflammatory arm, the antifibrotic arm had a significantly higher rate of seropositivity and antibody titer (Additional file 1: Table S1).

**Table 2:** Baseline characteristics and antibody titers among patients treated with anti-inflammatory treatment and matched control

	Anti-inflammatory Treatment (n = 29)	Matched Control (n = 29)	P value
Age, median (IQR), y	67 (55–73)	70 (56–72)	0.86
Male sex (%)	11 (38)	11 (38)	1.0
Anti-inflammatory treatment			
Antimetabolites and OCS	21	NA	
Rituximab	8		
Daily OCS dose (mg), median (IQR)	8 (5–8)		
Daily mycophenolate mofetil dose (g), median (IQR)	2 (1–2)		
Rituximab doses, median (IQR)	3 (2–4)		
Days post last rituximab treatment, median (IQR)	123 (36–147)		
IgG titer, median (IQR), Au/ml	39.60 (4.25–165)	970.10 (505–1926)	< 0.001
IgG titer GMT (SD)	23.70 ± 12.73	1006.93 ± 2.45	< 0.001
Seropositive (%)	14 (48.3)	29 (100)	< 0.001
Days post vaccination, median (IQR)	179 (168–184)	137 (122–158)	< 0.001

OCS: Oral glucocorticoid

**Fig. 1:**
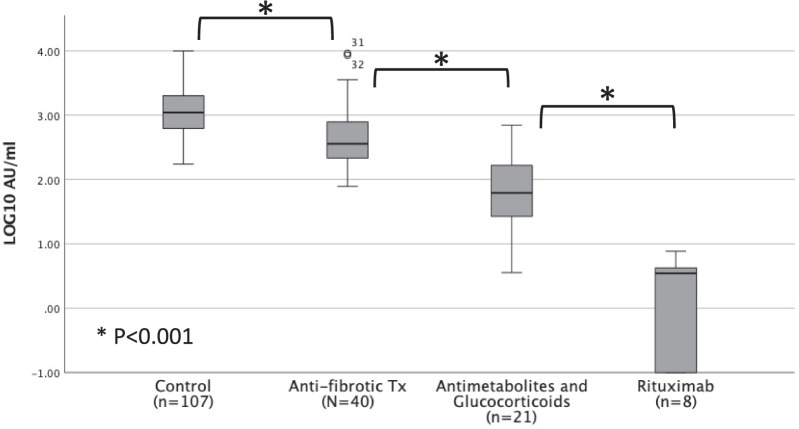
Antibody titer according to treatment type

Overall patients in the control group had the highest median antibody titer (1102, IQR 611–2034) followed by patients treated with antifibrotic drugs (361, IQR 207–811), antimetabolites and corticosteroids (61.8, IQR 21–214) and rituximab (3.5, IQR 0–4.3), overall difference and pairwise comparison were all significant (P < 0.001 in all between-group comparisons) (Fig. 1). Multivariate analysis showed that anti-inflammatory treatment significantly reduced the immune response while, age, antifibrotic therapy and the number of days post vaccination did not have a significant effect (Table 3). Covid-19 infection rates 6 months after antibody level analysis were 29% (31/107), 12.5% (5/40) and 43% (12/28) in the control, IPF, and non-IPF ILD groups, respectively (Additional file 1: Table S1).

**Table 3:** Multivariate analysis: Predictors for reduced humeral response

	OR	CI	P value
Age	− 13.77	− 31.62 to 4.08	0.13
Anti-fibrotic Tx	− 311.76	− 1002.82 to 379.29	0.37
Anti-inflammatory Tx	− 1287.21	− 1992.15 to (− 582.81)	< 0.001
Time from vaccine	− 1.01	− 10.51 to 8.48	0.83

## Discussion

**Fig. 2:**
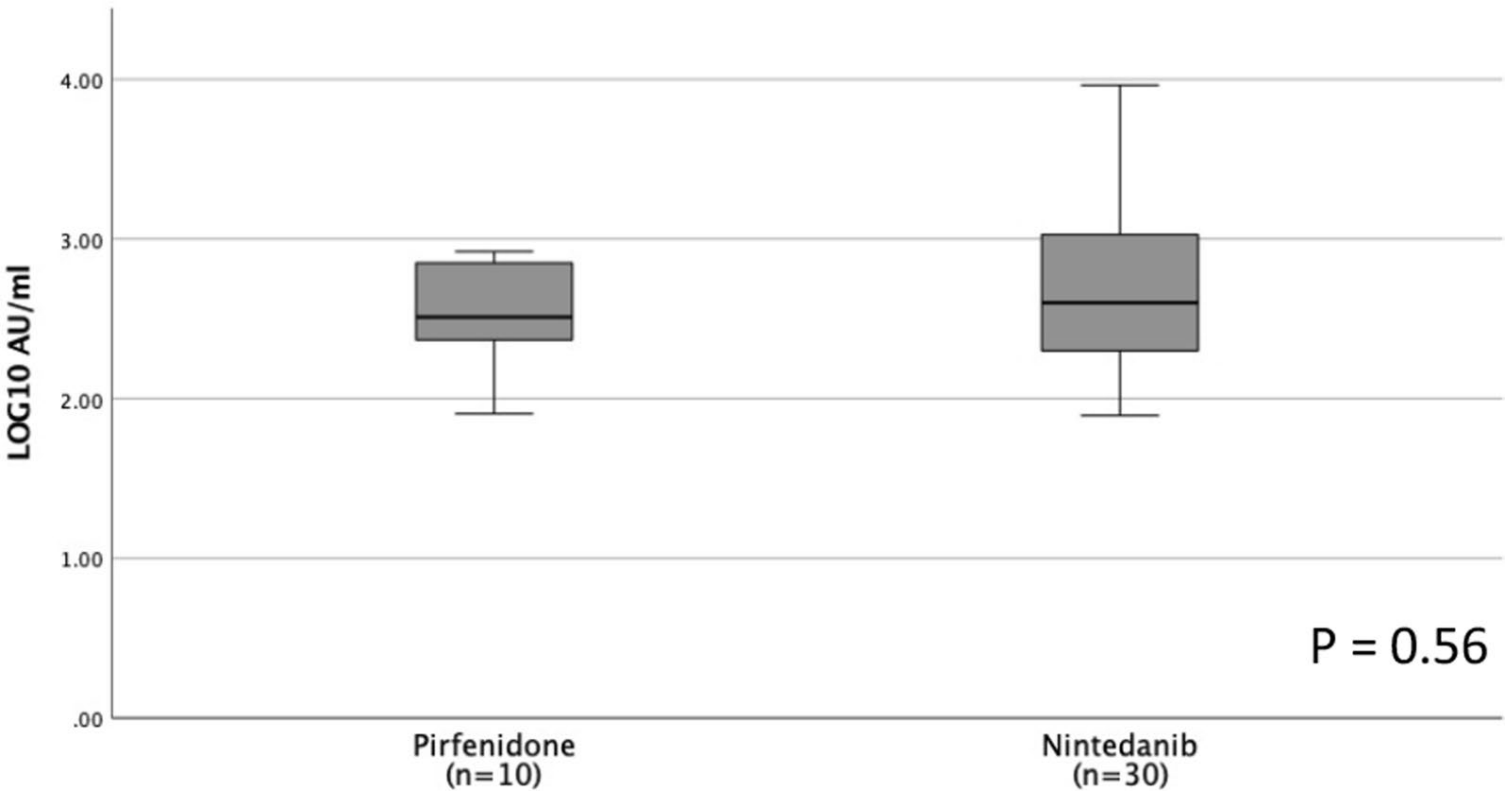
Comparison of S-IgG antibody titer in between patients treated with nintedanib vs pirfenidone

This study evaluated the humoral response among IPF patients treated with antifibrotic therapy and non-IPF ILD patients treated with anti-inflammatory treatment in comparison to an age and sex matched healthy control group, after 2 doses of the BNT162b2 SARS-CoV-2 vaccine. The study showed that IPF patients treated with antifibrotic therapy mount an adequate immune response to the BNT162b2 SARS-CoV-2 vaccine. However, with a reduced antibody titer in comparison to the control group. To our knowledge this is the first study to show that antifibrotic treatment enables a suitable immune response to the COVID-19 mRNA vaccine. The antibody titer in both treatment arms was evaluated 4–6 months after the second vaccine dose, at this time point the level of S-IgG antibodies is reduced in comparison to the early post vaccination period, since the antibody titer slowly declines after receiving the second vaccine dose [17]. Nevertheless, all patients in the anti-fibrotic group were seropositive at this time point, which further strengthens the conclusion that these patients mount and maintain an adequate immune response 4–6 months after vaccination. A recently published preprint that evaluated the impact of chronic disease on the humoral response after mRNA vaccine, found that ILD was an independent risk factor for reduced immunity independent of the patients medication use [5]. Furthermore, several trials have shown that in addition to their anti-fibrotic effect, both pirfenidone and nintedanib have an anti-inflammatory effect, which can interfere with humoral response. In pirfenidone by inhibition of dendritic cells, macrophages, neutrophils, eosinophils and T lymphocytes [18] and in nintedanib by blocking T cell activation [6, 19]. Although, we cannot ascertain whether the mildly reduced antibody titer in treated IPF patients was the result of the chronic interstitial lung disease or the antifibrotic treatment, it was most probably the result of both factors. Due to the differences in drug mechanism of action among the antifibrotic drugs, we also compared the titer of S-IgG antibodies in pirfenidone and nintedanib users and found no differences (Fig. 2). Finally, we evaluated the effect of different treatment regimens on the humoral response and showed that anti-inflammatory treatment with antimetabolites, glucocorticoids and rituximab causes a far greater reduction in immunity, in comparison to antifibrotic treatment. This further emphasizes the difference in mechanism of action of the antifibrotic drugs, which lead to improvement in clinical outcomes in IPF patients, in contrast to anti-inflammatory treatment [20].

The anti-inflammatory arm showed a significantly reduced humoral response in comparison to the control group. These results are in agreement with several studies that showed reduced humoral response in patients with inflammatory bowel disease, connective tissue disease and solid organ recipients, who were treated with anti-inflammatory therapy [2–4]. Patients treated with rituximab showed significant immunosuppression, even in comparison to other anti-inflammatory treatments. Comparable results were seen in similar trials, this profound immunosuppression should be taken under consideration when this drug is prescribed in the COVID-19 era [21–23].

Covid-19 infection rates 6 months post-analysis were significantly higher in the anti-inflammatory arm in comparison to the anti-fibrotic arm (Additional file 1: Table S1). Patient behavior (e.g., avoidance, repeated exposure as medical personnel) can dramatically affect the infection rates. Therefore, comparing the treatment arms, which include patients with chronic disease, to the control group, which includes healthy participants and medical personnel, is imperfect. Nonetheless, the IPF and non-IPF ILD groups include similar patients, and in that regard, a comparison of infection rate is of value and suggests that seropositivity lowers the risk of infection. Moreover, these data further emphasize the importance of vaccination in patients with ILD, who are at greater risk of both infection and death from COVID-19 [24–26].

This study has several limitations, there is a one-month difference in the time between the second vaccine dose and the serology test, between the control group and the anti-fibrotic and anti-inflammatory groups. Additionally, between the anti-fibrotic arm and the matched control group was a small yet statistically significant age difference. Nonetheless, we were able to show in the multivariate analysis that age and the number of days from the second vaccine did not influence the antibody titer (Table 3). The sample size was small, 40 patients in the antifibrotic arm and 29 patients in the anti-inflammatory arm. Nevertheless, this study was the first to describe IPF patients specifically and for this relatively rare disease the sample size allows for statistical analysis and meaningful conclusions. Finally, the study evaluated anti spike IgG antibodies and not neutralizing antibodies.

In conclusion, IPF patients treated with antifibrotic treatment mount an adequate immune response after 2 doses of the BNT162b2 vaccine and maintain 100% seropositivity rate 4–6 months after vaccination. However, the antibody titer was reduced in comparison to an age matched healthy control group. Among patients with non-IPF ILD, treated with anti-inflammatory therapy, 48% were seronegative 4–6 months after the second vaccine dose, moreover treatment with rituximab caused significant immunosuppression, even in comparison to other anti-inflammatory treatments.

## Supplementary Information

**Additional file 1: Table S1.** Baseline characteristics, antibody titers and COVID-19 infection rates among patients treated with anti-fibrotic and anti-inflammatory treatment.

## Data Availability

The datasets generated and/or analysed during the current study are available from the corresponding author on reasonable request.

## Abbreviations

COVID-19: Coronavirus disease 2019
ILD: Interstitial lung disease
IPF: Idiopathic pulmonary fibrosis
SARS-Cov-2: Severe acute respiratory syndrome coronavirus 2
S-IgG: Spike protein immunoglobulin G
