# Supplementary material for: Humoral response among patients with interstitial lung disease vaccinated with the BNT162b2 SARS-Cov-2 vaccine: a prospective cohort study
Source: Respir Res. 2022 Sep 1;23:226. doi: 10.1186/s12931-022-02155-x (PMC9433517; doi:10.1186/s12931-022-02155-x)
Supplement: Supplementary file 1 — Additional file 1: Table S1. Baseline characteristics, antibody titers and COVID-19 infection rates among patients treated with anti-fibrotic and anti-inflammatory treatment. [file 12931_2022_2155_MOESM1_ESM.docx]

Supplementary material

Table S1: Baseline characteristics, antibody titers and COVID-19 infection rates among patients treated with anti-fibrotic and anti-inflammatory treatment

|  | **Anti-inflammatory Tx**  **(n=29)** | **Anti-fibrotic Tx**  **(n=40)** | **P value** |
| --- | --- | --- | --- |
| **Age** | 63.9±12.51 | 71.03±8.00 | 0.01 |
| **Median (IQR) Au/ml** | 39.60 (4.25-165) | 361.10 (207-811) | <0.001 |
| **GMT** | 23.70±12.73 | 441.26±3.15 | <0.001 |
| **Adequate humoral response (%)** | 14 (48.3) | 40 (100) | <0.001 |
| **Time from 2^nd^ vaccine (days)** | 173.48±18.81 | 172.08±16.04 | 0.73 |
| **Infection rates six months post analysis (%)** | 12 (41.4) | 5 (12.5) | 0.006 |
